# CeO_2_-based catalysts with engineered morphologies for soot oxidation to enhance soot-catalyst contact

**DOI:** 10.1186/1556-276X-9-254

**Published:** 2014-05-23

**Authors:** Paolo Miceli, Samir Bensaid, Nunzio Russo, Debora Fino

**Affiliations:** 1 Department of Applied Science and Technology, Politecnico di Torino, Corso Duca degli Abruzzi 24, Torino 10129, Italy

**Keywords:** Soot oxidation, Diesel particulate filter, Ceria, Catalyst morphology

## Abstract

As morphology plays a relevant role in solid/solid catalysis, where the number of contact points is a critical feature in this kind of reaction, three different ceria morphologies have been investigated in this work as soot oxidation catalysts: ceria nanofibers, which can become organized as a catalytic network inside diesel particulate filter channels and thus trap soot particles at several contact points but have a very low specific surface area (4 m^2^/g); solution combustion synthesis ceria, which has an uncontrolled morphology but a specific surface area of 31 m^2^/g; and three-dimensional self-assembled (SA) ceria stars, which have both high specific surface area (105 m^2^/g) and a high availability of contact points. A high microporous volume of 0.03 cm^3^/g and a finer crystallite size compared to the other morphologies suggested that self-assembled stars could improve their redox cycling capability and their soot oxidation properties. In this comparison, self-assembled stars have shown the best tendency towards soot oxidation, and the temperature of non-catalytic soot oxidation has dropped from 614°C to 403°C in tight and to 552°C in loose contact conditions, respectively. As far as the loose contact results are concerned, this condition being the most realistic and hence the most significant, self-assembled stars have exhibited the lowest *T*_10%_ onset temperature of this trio (even after ageing), thus proving their higher intrinsic activity. Furthermore, the three-dimensional shape of self-assembled stars may involve more of the soot cake layer than the solution combustion synthesis or nanofibers of ceria and thus enhance the total number of contact points. The results obtained through this work have encouraged our efforts to understand soot oxidation and to transpose these results to real diesel particulate filters.

## Background

An increasing share of the automobile market has been gained by diesel engines on-board passenger cars, over the last two decades, as they are more fuel economic than gasoline vehicles. However, diesel engines entail a more challenging reduction of pollutant emissions. Particulate matter (PM) is a complex aerosol composed of nanosized carbonaceous particles (called soot) on which soluble hydrocarbons, sulphates and metals adhere through complex filtration and oxidation phenomena. These particulates have diameters that range from a few nanometers to hundreds of nanometers and beyond [1]. This means serious problems in terms of human respiratory diseases and environmental issues [2,3].

Driven by compulsory legislation, the reduction in PM emission is currently a technological challenge from both the engine and the catalyst points of view. In the past, many efforts were devoted to the development of catalytic diesel particulate filters (DPF), in order to achieve a cheaper and more effective solution than fuel-borne catalysts (FBC), which had proved to produce more pulmonary intrusion particles [4]. The DPF is a ceramic filter with alternate-plugged channels, in which the flue gases enter the open channels at the inlet, cross the porous ceramic wall of the channel, where soot particles are retained, and finally exit the filter from the neighbouring channels. The soot particles deposit in the pores of the ceramic walls and progressively form a soot layer on top of the wall, which is called *cake*[5]. The latter generates a drop in pressure across the filter, which becomes unsustainable for the engine; therefore, the cake periodically needs to be burned off, in order for the filter to regenerate. Regeneration is currently achieved through the post-injection of fuel from the engine [6,7], which causes a relevant fuel penalty for modern engines.

Currently, the combination of a trap with an oxidative catalyst is commonly adopted. This involves the deposition of noble metals on carriers with a high surface area, such as zeolites or γ-alumina, or those with redox properties, like ceria (CeO_2_) in pure or doped form [8,9]. It is common knowledge that rare earth metals, like ceria, are less expensive than classic noble metals and leave a lower transformation carbon footprint, which makes these materials more sustainable. Replacing noble metals with rare earth ones, or lowering the content of the former, would be a remarkable result in economic and environmental terms.

In this work, ceria-based catalysts have been investigated as active carriers to improve soot oxidation. In particular, three different morphologies have been proposed. Having redox properties, the Ce^4+^/Ce^3+^ cycle can store oxygen in lean conditions and then provide it in rich conditions to promote oxidation at the soot-catalyst interface [10]. This ability depends to a great extent on the intrinsic activity of the catalyst and on the properties of the reaction surfaces [11]. Redox-capable catalysts are developed by increasing oxygen mobility with vacancies, by exposing particularly active crystalline planes [12], or by enhancing the oxygen storage capacity (OSC) of catalysts in order to rapidly restore the oxidation state of the active metal following soot oxidation [13].

Another important fact is that soot oxidation is a solid-solid catalysis, and it is necessary to take into account the importance of the soot/catalyst contact conditions, which can basically be of two kinds: tight contact and loose contact. It has been demonstrated, in a real DPF, that loose contact takes place [14] and, in these conditions, the activity of the catalyst is not the only important feature: an engineered morphology has to be designed to achieve better results.

On the basis of this evidence, new morphologies were investigated in previous works [9,11], and in particular, a fibrous structure of the ceria-based carrier was proposed with the aim of maximizing contact between the catalyst and the soot particles. Despite their low specific surface area (SSA), these fibers in fact have a filamentous structure which enhances the number of soot-fiber contact points and, in some cases, show better performances than foamy or higher SSA nanopowders, obtained with the solution combustion synthesis (SCS) technique [9,11]. This proves that specific surface area is not the only important factor in solid-solid catalysis and that tailored morphologies can be achieved even with low specific areas.

This concept is extremely important, given the application field of these catalysts, which have to be layered on the surface of the DPF channels. A morphology that could intercept a higher fraction of the soot cake, with a better penetration of the catalytic layer inside the soot cake, would improve the regeneration phase.

As a result, a comparison of the three different ceria morphologies, namely the nanofibers, self-assembled stars and the nanopowders obtained by SCS, has been performed in the following study.

## Methods

### Synthesis

Three different synthesis techniques were adopted in this study:

▪ The CeO_2_ nanofibers were synthesized by means of the precipitation/ripening method [9,15]: starting from a 1 M aqueous solution of cerium (III) nitrate hexahydrate precursor (Sigma-Aldrich, St. Louis, MO, USA, 99%), the fibers were synthesized using a rotary evaporator and varying the NaOH/citric acid molar ratio. The residence time and conditions inside the evaporator led to different morphologies. A clear fibrous structure was obtained for a ratio of 0.8 at a constant temperature of 60°C for 6 h. One-hour drying at 110°C and calcination for 5 h in air at 600°C were performed. These processes did not cause the fibrous structure to collapse after the thermal treatment.

▪ The CeO_2_ self-assembled stars were prepared by mixing 0.2 M of cerium (III) chloride heptahydrate, 0.01 M of CTAB (both from Sigma-Aldrich) aqueous solutions and 80 mmol of solid urea. A hydrothermal treatment at 120°C for 12 to 24 h led to a precipitate, which was centrifuged, rinsed, filtered, dried at 60°C for 24 h and finally calcined at 600°C for 4 h [16]. The residence time inside the reactor in hydrothermal conditions affects the size and shape of these systems, as will be shown later on.

▪ The SCS was also used for the ceria catalyst preparation [17] in order to compare the foamy catalyst obtained with this technique with the above-mentioned alternative morphologies. In the SCS technique, a homogeneous aqueous solution of metal nitrates and urea is placed in an oven set at a constant temperature of 650°C. The solution quickly begins to boil and froth, and ignition then takes place. The exothermic reaction, due to urea combustion, provides the heat necessary for the endothermic transformation of nitrates into the desired oxide. The whole process is over in a few minutes, and the result is a foam that crumbles easily. In this case, the size and shape of the CeO_2_ structures were not tunable as in the other two cases, although a foamy structure and a moderate SSA were easily reached.

### Characterization

All the aforementioned CeO_2_ morphologies were characterized by means of X-ray diffraction (PW1710 Philips diffractometer, Amsterdam, The Netherlands, equipped with a Cu Kα radiation monochromator to check that the cerium oxide crystalline structure had been achieved and to estimate the average crystallite size via the Debye-Scherrer technique. A field emission scanning electron microscope (FESEM, Leo 50/50 VP Gemini column) was used to analyze the morphology of the CeO_2_ structures and to correlate it to its activity towards soot oxidation. A BET analysis (Micromeritics ASAP 2010 analyzer, Norcross, GA, USA) was conducted to evaluate the specific surface area of the catalysts and to perform a porosimetry analysis of the prepared catalysts. An ageing thermal treatment was performed for all three catalysts at 600°C for 5 h in order to have a better understanding of their reliability and performances under stressed conditions, namely when exposed to high temperatures for a certain period.

### Activity

Temperature-programmed combustion tests (TPC) were run to establish the oxidation activity of the catalysts, both in tight contact, in order to assess their intrinsic activity, and in loose contact, in order to evaluate their behaviour in more realistic conditions.

The tight contact was prepared by ball milling the catalysts and soot for 15 min at 240 rpm; this creates a intimate contact between the two phases and is helpful to discriminate the activity of the different morphologies. Only two 1 cm diameter agate balls were used instead of standard four to prevent breaking of the delicate micrometric structures during milling, as it had been noticed during the scanning electron microscopy (SEM) analysis, that severe mechanical stress could wreck such engineered morphologies. Loose contact was obtained by gently mixing the catalyst and soot with a spatula by hand for a minute. This technique, which is quick and easy but with reproducible results, simulates the real contact conditions for soot and a catalyst inside a DPF since the cake deposits on the filter channels without any external compaction force.

TPC runs were made with a PID-regulated tubular oven, into which a U-tube quartz reactor with the catalytic bed had been inserted. The temperature rose till 750°C at 5°C/minute, while 100 ml/min of 10% O_2_ (obtained by dilution of air with N_2_) was made to flow through a fixed bed of 5 mg of Printex-U synthetic soot (Degussa, Essen, Germany), 45 mg of catalyst and 200 mg of silica, according to the standard operating procedure described in [11], with the only difference being an increased amount of silica in the catalytic bed, to achieve a better temperature homogeneity. The CO/CO_2_ concentration in the outlet gas was measured via NDIR analyzers (by ABB). Each test was repeated three times to ensure reproducibility of the obtained results. The peak temperature, *T*_p,_ in the TPC plot of the outlet CO_2_ concentration was taken as an index of the catalytic activity. The onset (*T*_10%_) combustion temperature, defined as the temperature at which 10% of the initial soot is converted, was also considered in order to better discriminate between the intrinsic catalytic activities of the prepared catalysts. The half conversion temperature (*T*_50%_) was also taken into account. The onset temperature is important to rank the catalysts, according to the catalytic reaction; other phenomena (such as mass transfer or diffusion limitations) may in fact influence the performances of catalysts at higher conversion stages.

The modification to the inert silica content in the bed composition led to slightly different oxidation temperatures for the materials tested in [11], especially as far as the onset temperature was concerned. In fact, the higher dilution heat capacity of the here adopted silica bed was relevant, especially at the reaction onset, i.e. when the heat released by soot oxidation was not able to self-sustain the reaction, and therefore had most impact on the reaction rate itself. However, the catalyst ranking in loose and tight contact conditions obtained in [11] has here been confirmed, and it has been shown that the SA stars offer a major improvement over the other ceria morphologies developed in this work.

## Results and discussion

### Characterization

**Figure 1:**
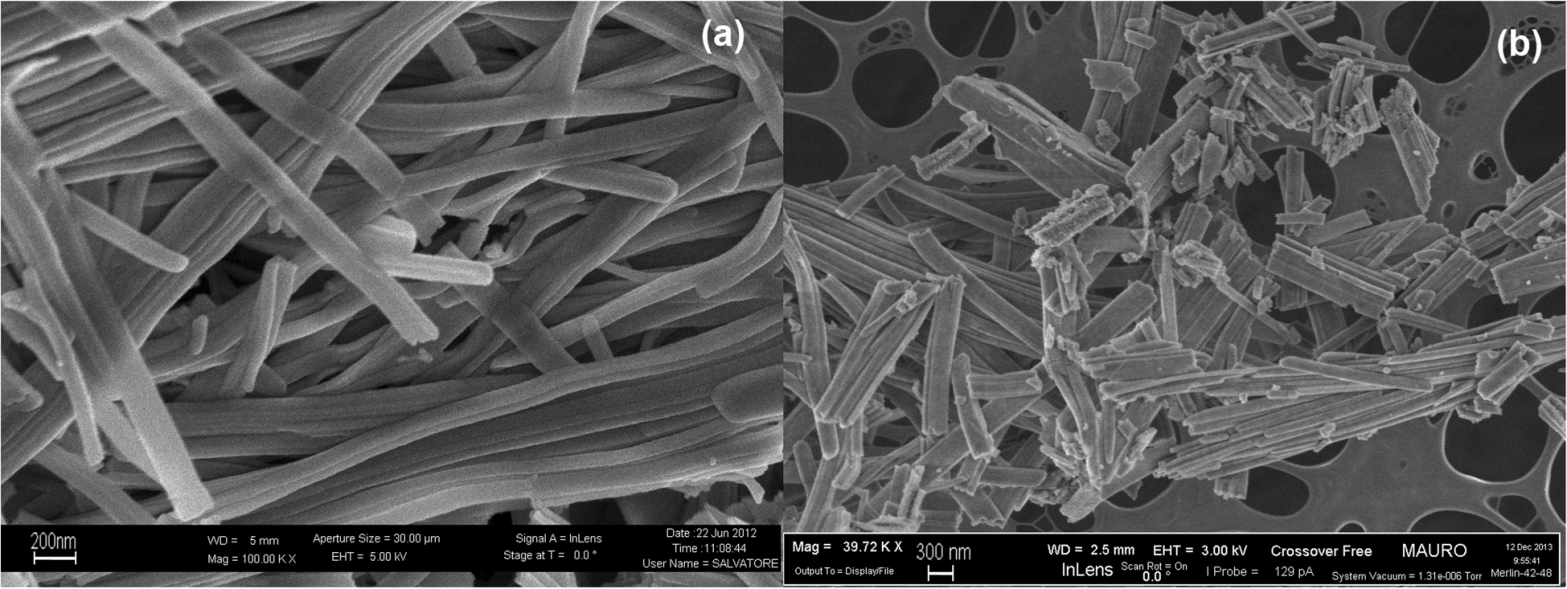
**FESEM images of the CeO**_**2 **_**nanofibers at × 100,000 (a) × 40,000 (b) level of magnifications.**

**Figure 2:**
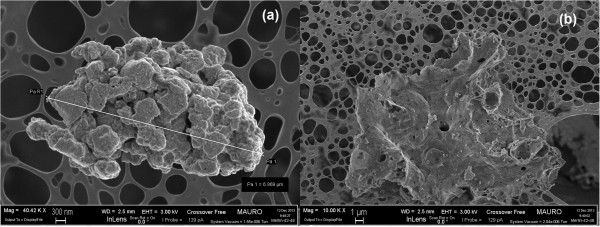
**FESEM images of the CeO**_**2 **_**SCS nanopowders at × 40,000 (a) × 10,000 (b) level of magnifications.**

The SEM analysis revealed the achievement of the desired morphologies sought for ceria. Figure 1 depicts the nanofiber ceria morphology, which shows a filamentous shape of the obtained structures, and a high aspect ratio, as already found in [9,11]. The three-dimensional network that is formed by the fibers has a high open porosity and is able to effectively come into contact with the soot particles in large number of points. Figure 2 reports the morphology of the nanopowders obtained by means of the SCS technique, which shows the rather uncontrolled shape of these catalysts. In this case, the aspect ratio is much smaller, and thus the maximum soot coverage of the particle, based on the catalyst weight, is lower.

**Figure 3:**
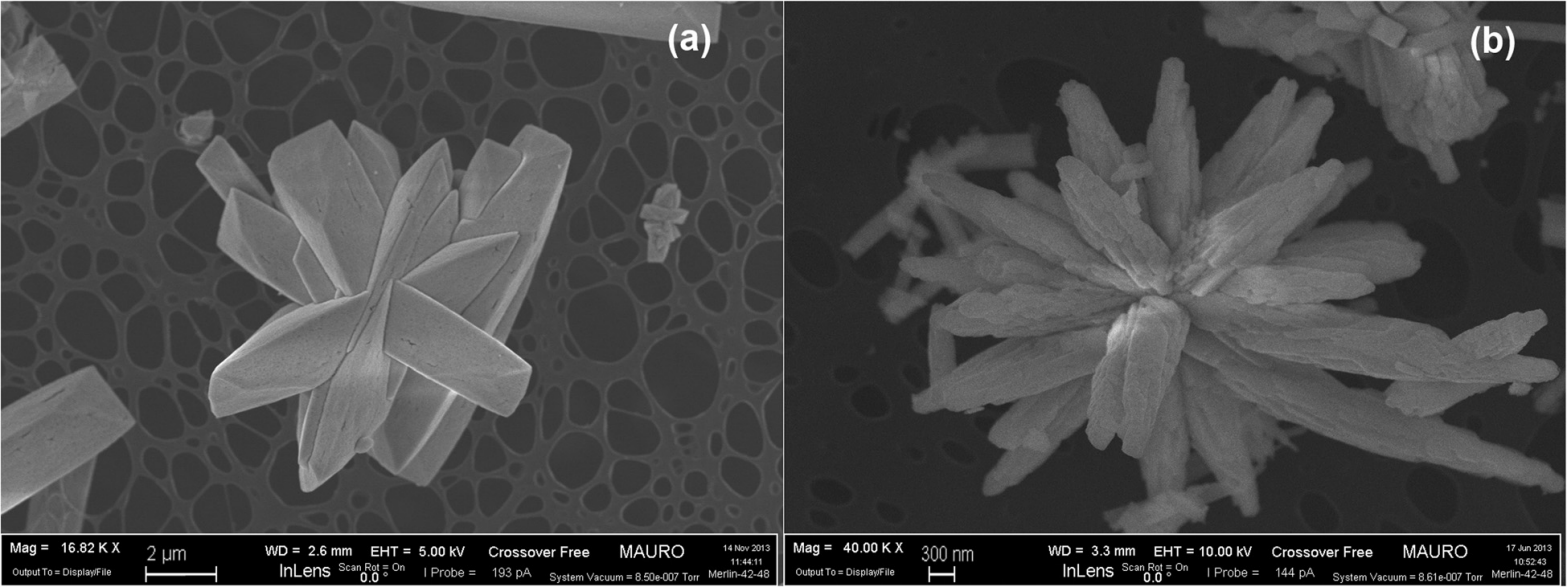
**FESEM images of the CeO**_**2 **_**SA-stars at 12 h (a) and 24 h (b) different residence times.**

**Figure 4:**
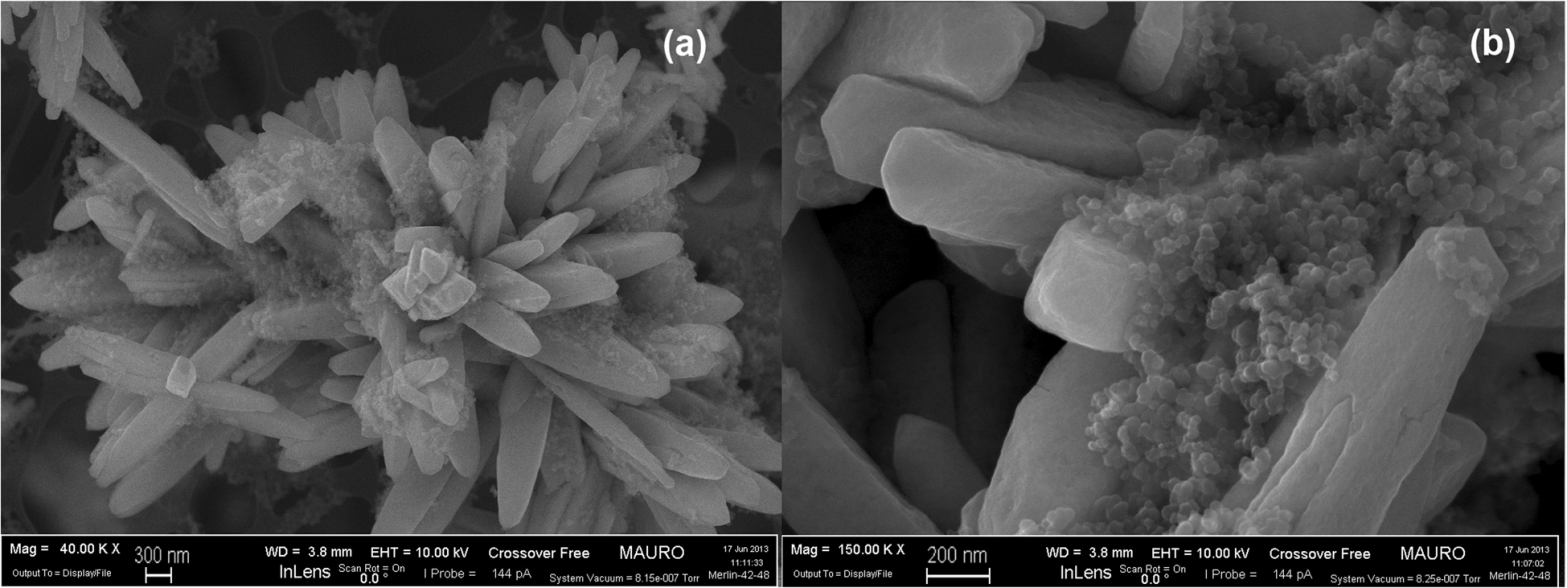
**FESEM images representing a loose contact mixture of CeO**_**2 **_**SA-stars and soot at × 40,000 (a) × 150,000 (b) level of magnifications.**

Finally, Figure 3 illustrates some details of a variety of self-assembled stars. The images show three micrometric star assemblies with different sizes and shapes, thus proving that the residence time in the reactor affects their final size (Figure 3a, 12 h; b, 24 h). This design offers a controlled and repeatable morphology, with a tridimensional shape constituted by individual rods (the fundamental elements that self-assemble into a star), which offer a concave space for soot intrusion. Soot-catalyst contact in loose conditions, before the TPC experiments, was observed by means of FESEM, and is depicted in Figure 4: it is possible to see that an effective soot penetration occurs, more so than would happen with a flat or convex morphology. This behaviour is desirable in the perspective of depositing such SA stars on the surface of the DPF channels as a carrier for noble metals or other active species: hence, an effective penetration of the soot cake through a relevant portion of the catalytic layer would increase the number of contact points between the soot particles and the catalyst itself, thus promoting catalyst activity. This would overcome the limitation of the catalytic layer obtained with *in situ* SCS [17], on the top of which the soot cake grows during soot filtration in the DPF: this generates a soot oxidation mechanism that only involves the interface between the catalyst layer and the soot cake.

**Figure 5:**
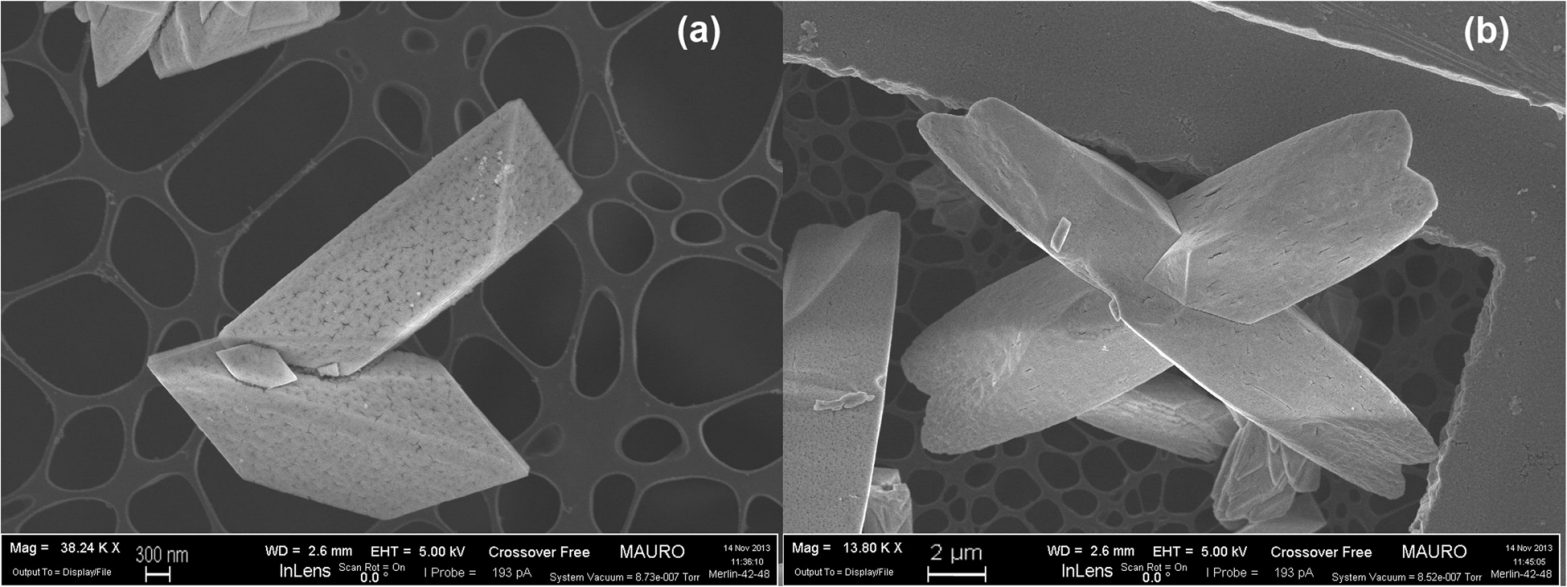
**FESEM images of CeO**_**2 **_**rods at × 38,000 (a) × 14,000 (b) level of magnifications.**

CeO_2_ has a fluorite cubic cell structure. It has been proved that hydrothermal treatments can expose unstable planes and turn the cube into an octahedron [12], whose tendency can be inferred from Figure 5. HRTEM investigations are needed to understand whether the obtained SA stars preferentially expose the most active ceria plains to soot oxidation, namely {310}, {100} and {110} even completely different structures [12,18]. These surfaces may be stabilized by defects (such as oxygen vacancy) or by adsorbed charge compensating species, and oxygen vacancies entail more oxygen mobility and availability for soot oxidation [19].

**Figure 6:**
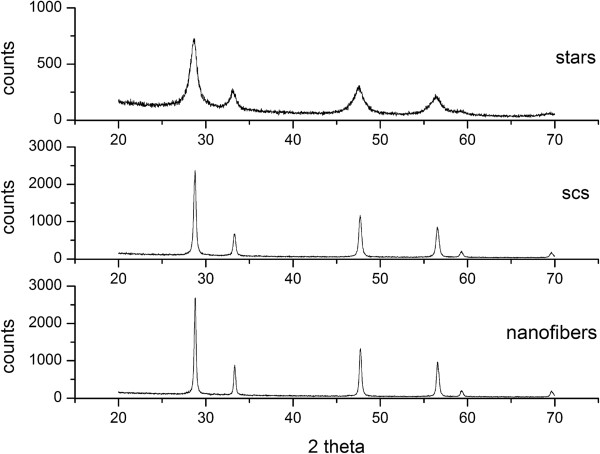
XRD spectra of the SA stars, SCS nanopowders and nanofibers.

The X-ray diffraction (XRD) analysis confirmed that all the catalysts belonged to the particular fluorite structure of CeO_2_ (*Fm-3 m*). From the comparison of the XRD spectra of the SCS ceria, fibers and SA stars, it is possible to appreciate a wider peak broadening in the star curves (Figure 6): according to the Debye-Scherrer theory, this entails finer crystallites for the SA stars. Moreover, the crystallite size distribution of the SA stars is the narrowest, as can be observed from Table 1, which reports the minimum, maximum and average crystallite size for the three morphologies. As the crystallites are smaller, the X-rays are diffracted over a much wider range of angles because of the large number of different crystalline domains and crystalline orientations. According to Kullgren et al. [19], the resulting smaller size of the SA star crystallites entails a greater presence of oxygen vacancies. The spectra of the SCS nanopowders and of the fibers are characterized by a lower number of crystalline domains, which entails fewer but larger grains. The smaller crystallite size in fact has an impact on the surface properties of the investigated catalysts.

**Table 1:** **Crystallite sizes of the CeO**_**2 **_**-based catalysts obtained by means of XRD analysis**

**Crystallite size [nm]**	**SCS**	**Nanofibers**	**SA stars**	**Aged SA stars**
Minimum	24	10	2	4
Maximum	55	100	10	23
Average	45	72	9	15

**Figure 7:**
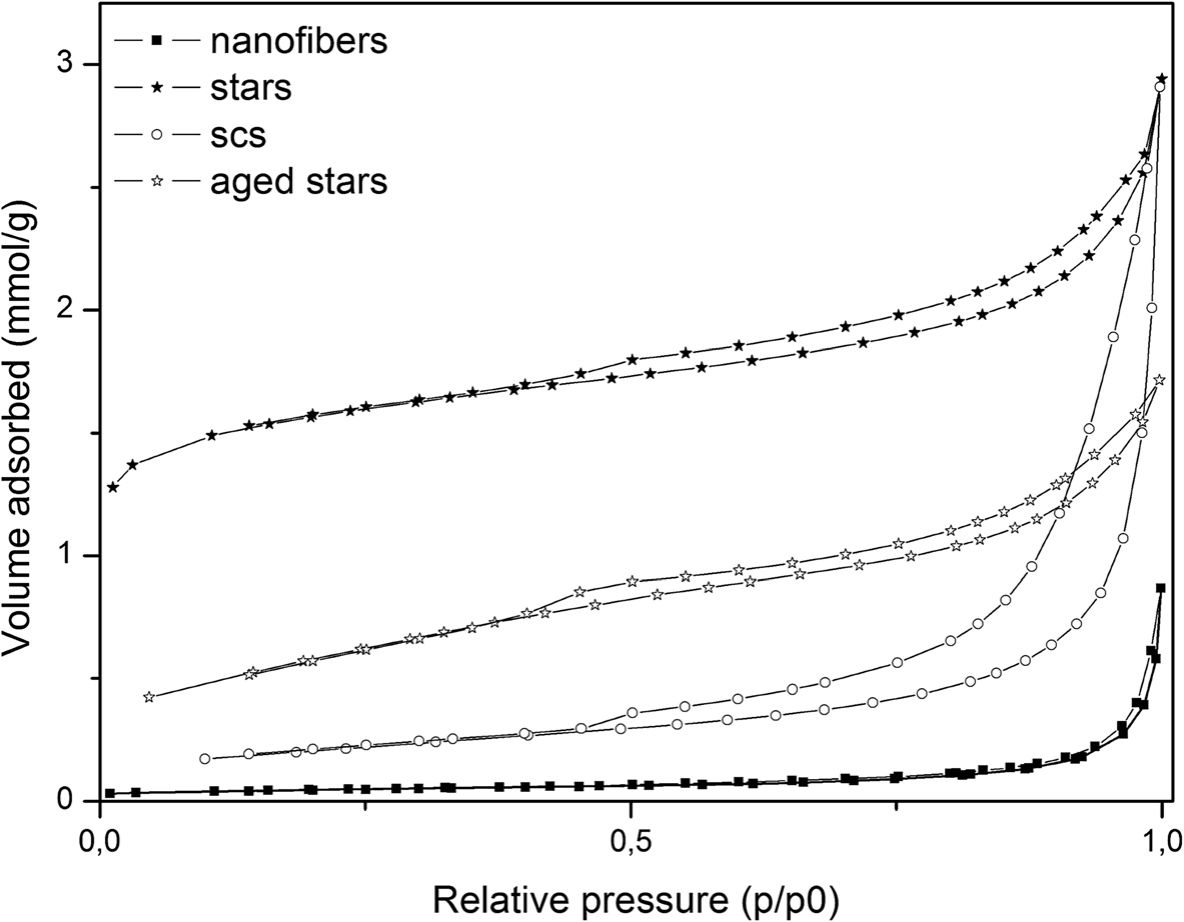
Porosimetry of the SA stars (fresh and aged), fresh SCS nanopowders and fresh nanofibers.

The BET measurements show, as reported in Table 2, that the SA stars have the highest SSA as-synthesized (being equal to 105 m^2^/g), even after ageing (50 m^2^/g). The porosimetries (Figure 7) on these catalysts revealed that the stars have a very high microporous volume (0.03 cm^3^/g). Conversely, the nanofibers are characterized by a very low specific area, while the ceria obtained with SCS lies somewhere in between the other two morphologies.

**Table 2:** **Specific surface area (SSA) of the CeO**_**2**_**-based catalysts obtained by means of BET analysis**

**BET (m**^**2**^**/g)**	**Fresh**	**Aged 5 h at 600°C**
SCS nanopowders	31	16
Nanofibers	4	1
SA stars	105	50

Recalling that soot oxidation depends on both the number of soot-catalyst contact points and on the availability of adsorbed oxygen at this contact point, it can be seen that the SA stars seem to have both features: they have the ability to maximize the contact between the soot and catalyst phase, as the fibers do, but they also have a much higher SSA, which entails a better activity at low temperatures (which depends on the oxygen coverage).

### Activity

All the prepared catalysts were tested under TPC runs towards soot oxidation, as previously described. Table 3 presents the tight contact results of the TPC runs for all of the catalysts, together with the Degussa soot blank run. The onset and half conversion values (*T*_10%_ and *T*_50%_) refer to the total conversion of soot to CO and CO_2_.

**Table 3:** Soot combustion activity results, under close and loose contact conditions, of the SA stars, SCS nanopowders and nanofibers

		***T***_**10% **_**(°C)**	***T***_**50% **_**(°C)**	***T***_**p **_**(°C)**
Soot		487	588	614
Nanofibers	Tight	383	439	445
	Loose	480	555	560
SCS	Tight	358	411	417
	Loose	483	562	562
SA stars	Tight	354	410	403
	Loose	435	543	552
Aged SA stars	Tight	381	453	465
	Loose	473	559	559

**Figure 8:**
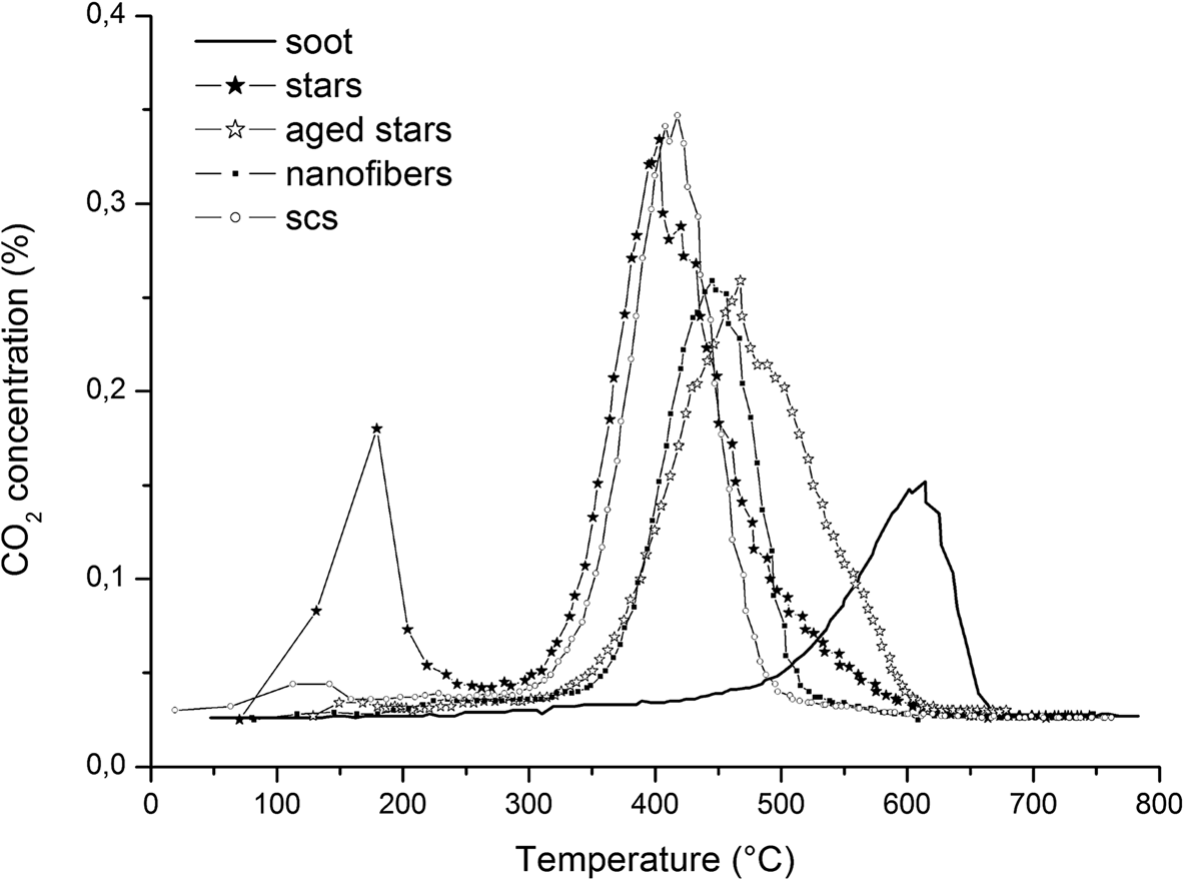
**CO**_**2 **_**concentration measured during the TPC runs, in close contact conditions.**

**Figure 9:**
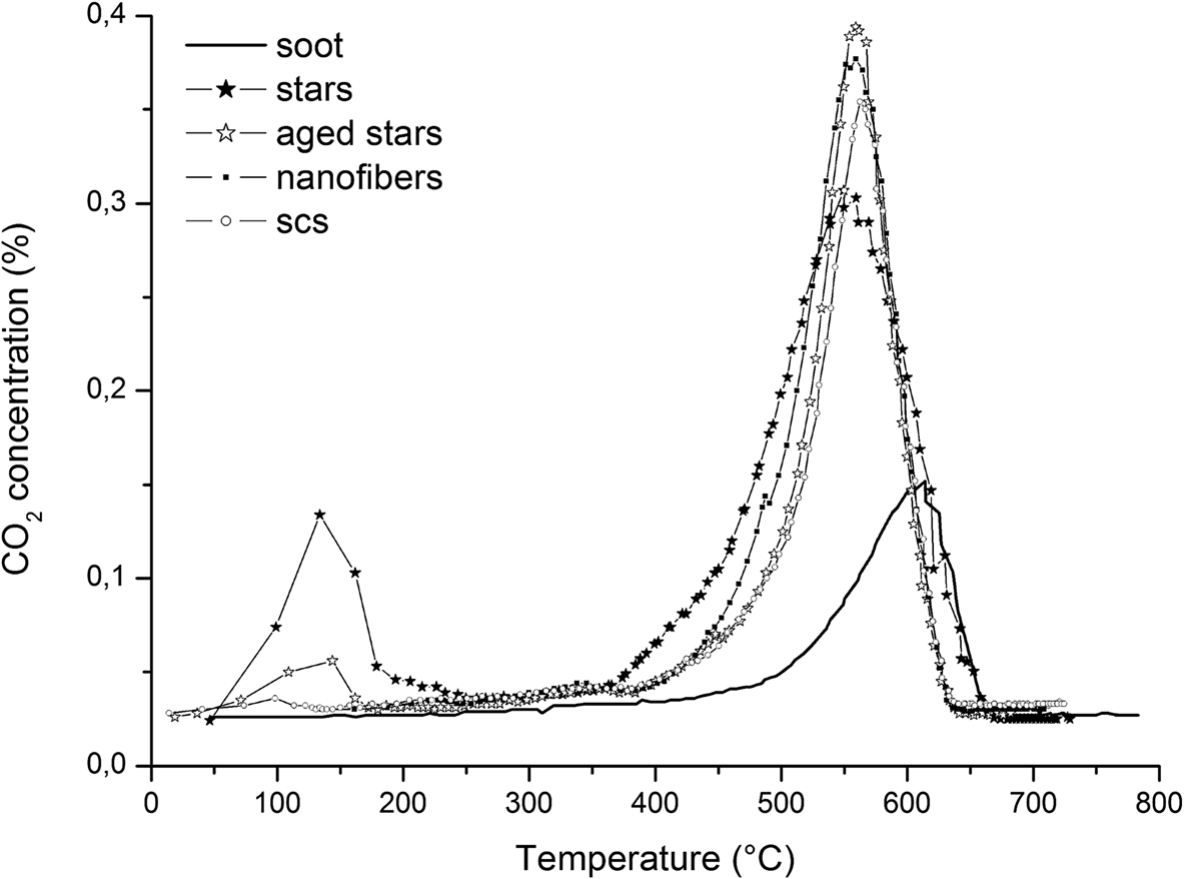
**CO**_**2 **_**concentration measured during the TPC runs, in loose contact conditions.**

The curves of the CO_2_ concentration are depicted in Figure 8 for all of the mentioned catalysts in tight contact conditions, while Figure 9 refers to the loose contact ones. SA stars and SCS nanopowders show the best performances in tight conditions, in terms of both *T*_10%_ and *T*_50%_, although the activity of SA stars decreases at higher temperatures. In tight contact, the mechanical force generates a particularly close contact between the soot and the catalyst, thus the advantages of the morphology are less important.

Conversely, in loose contact conditions, the morphology plays a more relevant role: the nanofibers, despite the almost null SSA, exhibit an almost equivalent activity to that of the SCS powders. This behavior, which was also obtained in [11], is here confirmed; this is further evidence that the BET alone cannot explain the activity of the soot oxidation catalytic reaction and that the contact between soot and the catalyst should be promoted. As far as the SA stars are concerned, their performance is much better than that of the other two catalysts, especially at low temperatures: in fact, the high porosity of the catalyst provides more adsorbed oxygen to the contact points between the soot and the catalyst, which is likely to be in a sufficient amount to fully exploit this oxygen availability. As far as the aged catalyst tests are concerned, it is worth mentioning that the lower SSA penalizes *T*_10%_, but *T*_50%_ still remains within the range of the other fresh catalysts.

A low temperature peak in the CO_2_ concentration (around 140°C) is evident in all the star-related curves. This peak is not connected to soot combustion. A tailored set of consecutive temperature-programmed desorption (TPD) runs was run to prove that the CO_2_ produced at low temperature is due to the desorption of CO_2_ from the inner nanoporosity of the self-assembled stars: in the first TPD, a fresh catalyst, previously exposed to air, was heated to 200°C in N_2_, and the CO_2_ desorption peak was recorded. The same catalyst was then cooled down in N_2_ and heated again in N_2_ to 200°C: in this case, no CO_2_ was noticed. The CO_2_ peak recorded at 140°C was therefore clearly attributable to the desorption of the CO_2_ formerly present in the air and was greater for the SA stars as they are characterized by the highest SSA.

**Figure 10:**
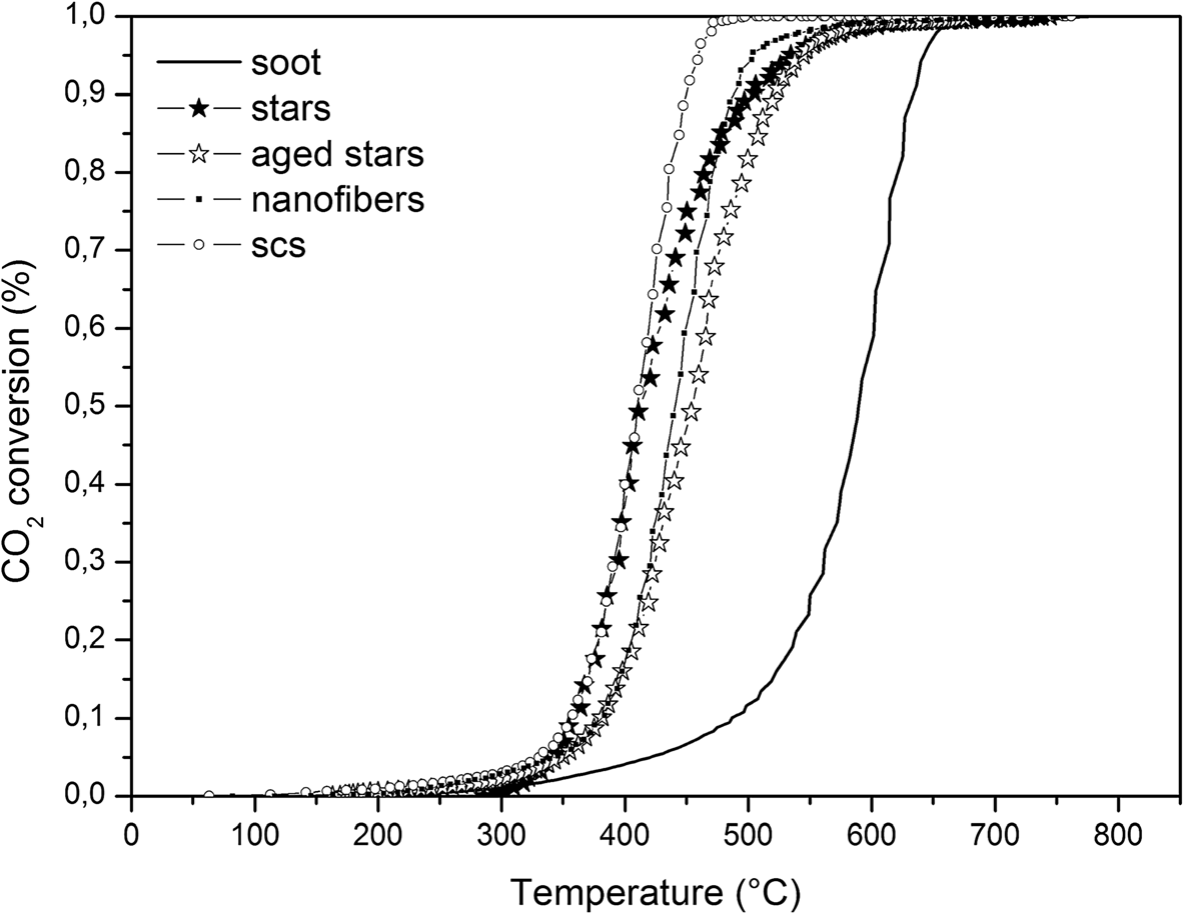
Total soot conversion in tight contact conditions.

**Figure 11:**
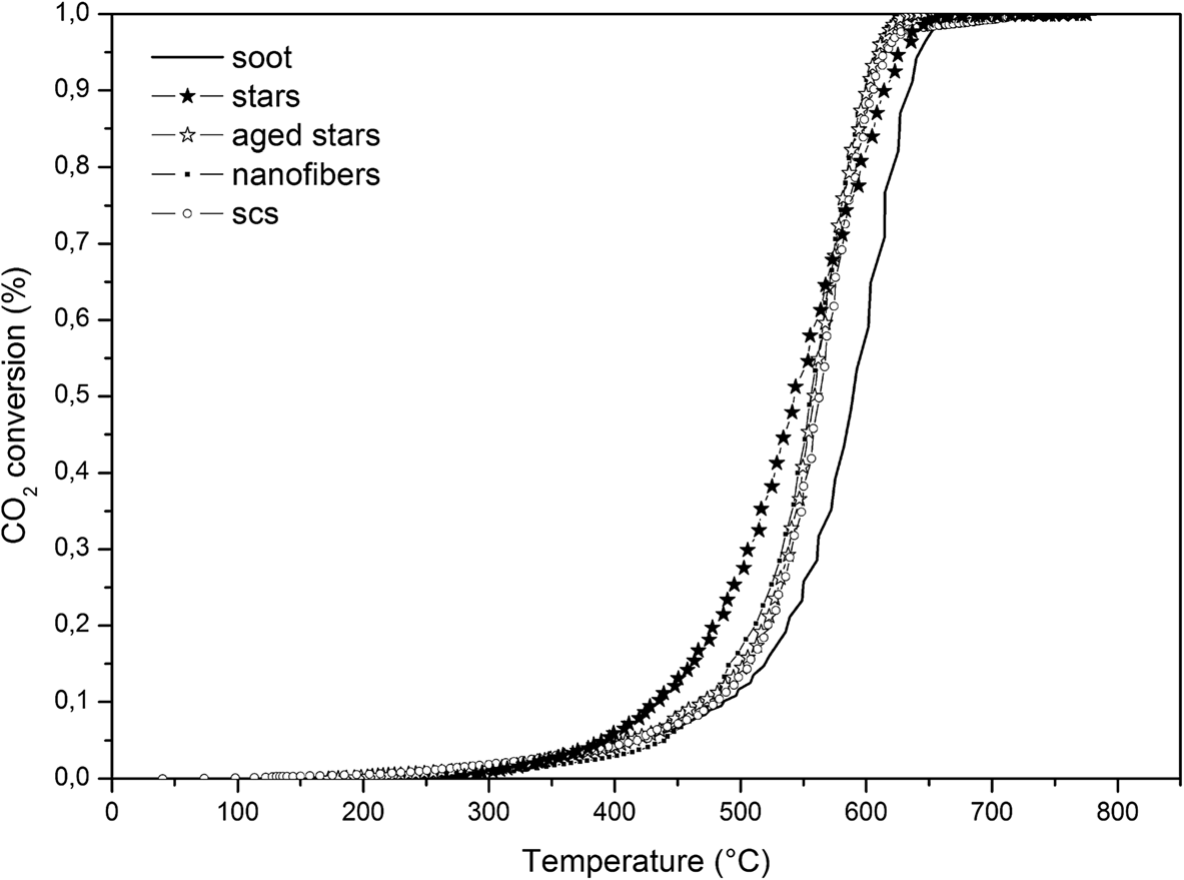
Total soot conversion in loose contact conditions.

Figures 10 and 11 show the total soot conversion curves, in tight and loose contact conditions, respectively. In particular, both plots highlight the higher activity of SA stars towards soot-burning ignition (*T*_10%_), but the performances decrease compared to SCS and nanofibers in the very last stage of the total oxidation. This behaviour may be due to the higher number of oxygen vacancies in the SA stars. In fact, the more oxygen vacancies, the more Ce^3+^ in the fluorite structure and, as the reduction of the remaining Ce^4+^ continues, the larger ionic radius of Ce^3+^ results in a change in the surface relaxation and modifies oxygen mobility, especially at higher temperatures [18]. Further HRTEM and OSC studies are needed to prove it.

## Conclusions

Three different types of ceria catalysts have been synthetized and compared for soot oxidation using TPC runs: SCS, with an uncontrolled morphology, and two engineered design ones, nanofibers and self-assembled stars.

The purpose was to create a catalytic layer in DPF that would be able to entrap soot particles in several active points and enhance oxidation for a fast and cheap regeneration of the filter. Several TPC runs have been conducted, in both tight and loose contact mode, to investigate the contact points of all the three catalysts.

In previous works [9,11], it was proved that engineered catalyst morphologies give better results towards soot oxidation than unstructured ones, and it was therefore decided to continue developing this idea and try and remove any drawbacks. A new morphology, with a star-like shape of micrometric size, was developed. It was deduced, from the TPC runs results, that SA stars give better results than the other catalysts, especially in loose conditions. In spite of their micrometric size, SA stars are nanostructured and have finer crystallite size: this entails a much higher BET area, greater availability of oxygen vacancies, more efficient redox cycles and, therefore, a higher oxidative capability.

Further investigations are needed to improve both the morphology and its effective deposition inside the DPF in order to improve the cake oxidation within the filter itself.

## Competing interests

The authors declare that they have no competing interests.

## Authors' contributions

PM participated in the design of the study, carried out all the experimental tests and helped to draft the manuscript. SB conceived the study and participated in its design and revised it critically for its important intellectual content. NR revised it methodically for its important chemical content. DF participated in the interpretation of the data, revised the article critically for its intellectual content and gave final approval of the version to be published. All the authors read and approved the final manuscript.
